# Development of Stable CHO-K1 Cell Lines Overexpressing Full-Length Human CD20 Antigen

**DOI:** 10.61186/ibj.27.5.269

**Published:** 2023-07-08

**Authors:** Niloufar Mohammadkhani, Azam Rahimpour, Reyhaneh Hoseinpoor, Masoumeh Rajabibazl

**Affiliations:** 1 Department of Clinical Biochemistry, School of Medicine, Shahid Beheshti University of Medical Sciences, Tehran, Iran;; 2 Cellular and Molecular Biology Research Center, Shahid Beheshti University of Medical Sciences, Tehran, Iran;; 3 Department of Tissue Engineering and Applied Cell Sciences, School of Advanced Technologies in Medicine, Shahid Beheshti University of Medical Science, Tehran, Iran;; 4 Department of Biotechnology, School of Advanced Technologies in Medicine, Shahid Beheshti University of Medical Sciences, Tehran, Iran

**Keywords:** CD20, CHO cells, Immunotherapy, Lentiviral vector

## Abstract

**Background:**

CD20 is a differentiation-related antigen exclusively expressed on the membrane of B lymphocytes. CD20 amplification is observed in numerous immune-related disorders, making it an ideal target for immunotherapy of hematological malignancies and autoimmune diseases. MAb-based therapies targeting CD20 have a principal role in the treatment of several immune-related disordes and cancers, including CLL. Fc gamma receptors mediate CD20 internalization in hematopoietic cells; therefore, this study aimed to establish non-hematopoietic stable cell lines overexpressing full-length human CD20 antigen as an in vitro model for CD20-related studies.

**Methods:**

*CD20* gene was cloned into the transfer vector. The lentivirus system was transfected to packaging HEK 293T cells, and the supernatants were harvested. CHO-K1 cells were transduced using recombinant viruses, and a stable cell pool was developed by the antibiotic selection. *CD20* expression was confirmed at the mRNA and protein levels.

**Results:**

Simultaneous expression of GFP protein facilitated the detection of CD20-expressing cells. Immunophenotyping analysis of stable clones demonstrated expression of CD20 antigen. In addition, the mean fluorescence intensity was significantly higher in the CD20-CHO-K1 clones than the wild-type CHO-K1 cells.

**Conclusion:**

This study is the first report on using second-generation lentiviral vectors for the establishment of a non-hematopoietic cell-based system, which stably expresses full-length human CD20 antigen. Results of stable CHO cell lines with different levels of CD20 antigen are well suited to be used for CD20-based investigations, including binding and functional assays.

## INTRODUCTION

CD20 is a non-glycosylated phosphoprotein and the first identified differentiation-related antigen exclusively expressed by B lymphocytes^[^^1^^]^. As a member of the MS4A family, CD20 antigen contains two extracellular domains^[^^2^^]^, which the long arm of chromosome 11 codes its sequence in human. The expression of CD20 molecule on B lymphocytes starts from pre-B cell linage, and it is maintained throughout lymphocyte differentiation, before terminal differentiation of B-cells into plasma cells^[^^3^^-^^5^^]^. Evidence has revealed that calcium homeostasis is altered by the involvement of CD20 in multiple downstream signaling pathways, indicating that CD20 function is beyond being merely a calcium channel^[^^6^^]^. 

MAbs have become a novel therapeutic strategy against various human maladies, including cancers, autoimmune disorders, and severe viral diseases^[^^7^^,^^8^^]^. CD20 blockade using mAbs has shown the critical role of CD20 in maintaining the B-cell compartment, B-cell differentiation, and proliferation-initiating events^[^^4^^,^^9^^,^^10^^]^. Pathways, which regulate adhesion and migration processes in cancerous B lymphocytes^[^^11^^]^, are upregulated through the stimulation by micro-environmental factors in lymph nodes^[^^12^^]^. Furthermore, CD20 amplification is observed in a broad number of B-cell hematological malignancies and autoimmune diseases, making CD20 an ideal target for immunotherapy in such disorders^[^^3^^,^^13^^-^^15^^]^. Anti-CD20 mAb-based therapies are among the most successful and effective targeted therapy approaches exploited in the treatment of several immune-related disorders and cancers, including CLL^[^^15^^,^^16^^]^. The first FDA-approved anti-CD20 mAb, rituximab, pioneered cancer therapy (1997)^[^^13^^]^, and then ofatumumab emerged in 2009, followed by obinutuzumab in 2013. As a treatment for the primary progressive form of multiple sclerosis, ocrelizumab was developed in 2017; this medication was able to foster immunotherapy in related diseases^[^^15^^,^^17^^]^. Nonetheless, downregulation or loss of CD20 on the targeted B cells can reduce the therapeutic efficacy of these strategies^[^^3^^,^^18^^,^^19^^]^ . In fact, reduction of cell-surface CD20 antigens results in decreased anti-CD20 mAbs binding and Fc-mediated effector functions^[^^5^^]^. 

The internalization of the complex formed between the rituximab and CD20 was examined by Beers and colleagues^[^^18^^]^. Beum et al*.*^[^^20^^]^ have also confirmed the internalization of the bound rituximab and ofatumumab antibodies in primary CLL cells. Immune cell inhibitory antibody receptors (FcγRIIb) are expressed on immune cells and govern most of mAbs efficacy after treatment^[^^21^^,^^22^^]^. It has been indicated that the FcγRIIb could mediate the internalization of mAbs-CD20 complex, and its degradation causes resistance to rituximab and ofatumumab as type I anti-CD20 mAbs^[^^23^^-^^26^^]^. Therefore, establishment of CD20 expressing non-hematopoietic cells could provide a suitable model system for various researches who work on CD20 molecule and mAbs targeting CD20, as well as in experiments designed to investigate the biological mechanisms involved in CD20 loss from B lymphocytes. 

In this study, a lentiviruses-based viral vector was used for stable expression of CD20 in CHO-K1 cells. Lentiviral vectors are efficient gene transfer vehicles for both dividing and non-dividing mammalian cells and can be utilized for rapid, scalable and high-level production of soluble and surface proteins^[^^27^^,^^28^^]^. Recently, these vectors have been employed in basic molecular research to carry components like guide RNAs for the CRISPR-Cas9 genome editing technologies^[^^29^^]^. They can enter the nucleus through the nuclear pore complex and are able to integrate the transgene into the genome of host cells, resulting in stable expression of the gene of interest^[^^13^^,^^24^^]^. Herein, we recruited second-generation lentiviral vectors to establish stable CHO cell lines expressing full-length human CD20 antigen. Schematic representation of the study is shown in Supplementary Figure 1. 

## MATERIALS AND METHODS

Cell lines and cell cultures

Raji as CD20-positive hematopoietic cells, HEK293T, and CHO-K1 cell lines were obtained from the Pasteur Institute of Iran (Tehran, Iran). HEK293T and CHO-K1 ce lls were respectively maintained in DMEM high glucose and DMEM F12 (both from Thermo Fisher Scientific, USA), and Raji cells were maintained in RPMI 1640 (PAN-Biotech, Germany). All the cultures were supplemented with 10% FBS (Gibco, Germany) and 100 IUmL^-1^ of penicillin and 100 µgmL^-1^ of streptomycin (both from Sigma Aldrich, Germany) and maintained in the presence of 5% CO_2 _at 37 °C.

Plasmids

The second-generation HIV-1-based lentiviral packaging system was used in this study. The system included (Ι) a copGFP-encoding transfer vector (pCDH; Systems Bioscience, US), (ΙΙ) a helper plasmid (pSPAX; Addgene 12260, Addgene, USA) expressing the viral *gag* and *pol* genes, which encode viral structural proteins and the reverse transcriptase and integrase, respectively^[^^25^^]^, and (ΙΙΙ) an envelope plasmid containing VSV-G (pMD2.G; Addgene # 12259, Addgene), which expresses the vesicular stomatitis virus envelope glycoprotein^[^^23^^,^^24^^]^.

Antigen sequences

The sequence of human *CD20* gene was acquired from NCBI databases (http://www.ncbi.nlm.nih.gov/ protein). Homo sapiens *MS4A1 *gene has three variants with accession numbers of NM_152866.3, NM_152867.2 and NM_021950.3 for variants 1 to 3, respectively. All three variants encode the same protein, and their difference is in the UTR area. Primer designing was performed for the coding area.

Cloning of CD20 fragment in pCDH expression vector

The blood sample was collected from a 77-year-old male CLL patient and transferred to EDTA-containing vials. PBMCs of the sample were isolated using Ficoll by a density gradient centrifugation method^[^^24^^]^. The cDNA was synthesized (M-MLV Reverse Transcriptase Kit, Yekta Tajhiz, Iran) from DNase-treated RNA following total RNA extraction from PBMCs (RiboEx TM kit, GeneAll, Korea). Then *CD20* gene was amplified by PCR. The forward and reverse oligonucleotide primers, shown in Table 1, were synthesized by Genefanavaran Company (Tehran, Iran). The PCR temperature condition and cycle numbers were as follows: (1) 95 °C for 10 min, (2) 95 °C for 30 s, (3) 61 °C for 30 s, (4) 72 °C for 30 s, and (5) 72 °C for 10 min. Cycles 2-4 were repeated 30 times. The PCR product was digested with *Bam*HI and *Xba*I (Thermo fisher scientific, Germany), in which their restriction sites were respectively added to the 5ʹ and 3ʹ ends of the gene. The cloning of constant fragment of CD20 antigen performed in pCDH vector digested with the two restriction enzymes. The pCDH vector contained ampicillin and puromycin resistance genes and encoded copGFP reporter gene. The transfer vector pCDH-CMV-MCS-EF1-GFP-T2A-Puro comprised the EF-1α and the human CMV major immediate-early promoter/enhancer.

Lentivirus production

Lentiviruses were generated using calcium phosphate-mediated transfection of packaging cells (Takara Bio, USA) as previously described^[^^30^^,^^31^^]^. One day prior to transfection, 3 × 10^5^ HEK 293T cells were seeded in each well of a six-well plate (SPL, South Korea) in 2 mL of DMEM high glucose containing 10% FBS, 100 IUmL^-1^ of penicillin, and 100 µgmL^-1^ of streptomycin. The next day, the cells were co-transfected with helper plasmid psPAX:helper plasmid pMD2G:LV transfer plasmid 1:1:2^[^^32^^]^. The CaPi-DNA co-precipitation containing 3 µg of plasmid DNA in 200 µL of solution volume was then added dropwise to the cells cultured in 1.8 mL of serum and antibiotic-free medium. At 4 h post transfection, the medium was replaced with 2 mL of a fresh DMEM high glucose comprising the same content of the DMEM mentioned above. The medium was harvested at 48 h and 72 h post transfection. The harvested medium was stored and pre-cleaned via centrifugation at 1500 ×g for 5 min and then filtered through a 0.45-µm membrane to remove cell debris. Viruses were overloaded on a sucrose-containing buffer and purified with centrifugation at 10000 ×g at 4 °C for 4 h^[33]^. Concentrated viruses were used immediately or stored at -20 °C. Successful transfection of the cells was evaluated through monitoring GFP expression in the transfected cells using fluorescence microscopy. 

***Titration of lentiviral vector stocks***

According to a modified protocol, the lentiviral vector titer was ascertained in CHO-K1 cells^[^^34^^]^. At 24 h prior to infection, CHO cells were seeded in 24-well plates at 1.5 × 10^5^ cells/well in 500 µL of DMEM/F12 (10% FBS, 100 IUmL^-1^ of penicillin, and 100 µgmL^-1^ of streptomycin) and incubated in the presence of 5% CO_2_ at 37 °C overnight. The next day, the medium was removed, and the cells in each well were transduced with serial dilutions of the LV stocks in a final volume of 500 µL of the serum and antibiotic-free medium in the presence of polybrene (hexadimethrine bromide; final concentration of 10 μgmL^-1^; Sigma Aldrich, Missouri, USA). At 24 h post infection, the medium was replaced with a fresh DMEM/F12 comprising the content similar to the above-mentioned DMEM. CHO-K1 cells were then incubated for another 48 h, and LV titer was calculated according to the provided equation^[^^35^^]^.

Titer (transduction unit mL^-1^) = ((% of GFP^+^ cells × cell number) ÷ (supernatant volume)) × dilution factor

**Table 1:** Primer sequences designed for the RT-PCR analysis

**Sequence**	**Primers**	**Restriction enzyme**
CD20	Forward: 5ʹ-ACTCA**TCTAGA**GCTAGCGAGCAAAATGACAACACCCAG -3ʹ	*Xba*I
	Reverse: 5ʹ- ACTAT**GGATCC**GAAATCACTTAAGGAGAGCTGTC -3ʹ	*Bam*HI

Bold regions indicate restriction sites on the primer sequences.

Transduction

To perform kill curve analysis, 2 × 10^5^ CHO-K1 cells were seeded in a 24-well plate in duplicate (SPL, South Korea) and cultured at different concentrations of puromycin (0, 2.5, 5, 7, and 10 µgmL^-1^) for two weeks. The medium was changed every 48 h. The lowest

puromycin concentration that caused massive cell death in 3-4 days as well as killed all cells in two weeks, was selected as the selection dose. CHO cells were transduced with lentivirus stocks in the presence of polybrene. Briefly, 10^5^ cells were seeded in 24-well plates in 500 µL of complete medium at a day before transduction and incubated in the presence of 5% CO_2_ at 37 °C overnight. The next day, cells were incubated with LV-containing and antibiotic-free medium in a final volume of 500 µL with the multiplicities of infection of 1 for 24 h, and then the medium was replaced with a fresh DMEM/F12 containing 10% FBS, 100 IUmL^-1^ of penicillin, and 100 µgmL^-1^ of streptomycin. Then the cells were incubated for 3-4 days before adding puromycin. For the generation of stable cell pool, transduced cells were cultured with selection-medium (6 µgmL^-1^ of puromycin) for two months. 

RT-PCR analysis 

Expression of *CD20* gene was examined using RT-PCR method following antibiotic selection. The total RNA of the cells was extracted from stable pool using the RiboExTM kit (GeneAll) according to the manufacturer’s instructions, and cDNA was synthesized from DNase-treated RNA using M-MLV Reverse Transcriptase Kit (Yekta Tajhiz). The primer sequences for the detection of the integrated gene of interest, temperature condition, and cycle numbers were the same as mentioned above in section 2.4.

Immunocytochemistry

Wild-type CHO-K1 cells and stable CHO-K1-CD20 cells as negative and test groups, respectively, were seeded a day before the examination at a density of 15 × 10^3^ cells/well in a 96-well plate in triplicate and incubated in the presence of 5% CO_2 _at 37°C overnight. The next day, 300 µL of ice-cold methanol solution was added to each well and incubated at -20 °C for 20 min to fix cells. Wells were then washed with PBS and blocked with 4% skim milk at room temperature for one hour. Following washing, 100 µL of anti-CD20 ofatumumab antibody that was previously expressed and purified in CHO cells was added^[^^36^^]^, while PBS as negative control was added to a group of CHO-K1-CD20 cells. The wells were incubated at 37 °C for 1.5 h and shaken at 70 rpm, washed three times using PBS (pH 7.2), and added to a secondary anti-human IgG antibody labelled with horseradish peroxidase. The wells were further incubated at 37 °C for 1.5 h, and 100 µL of TMB (3,3',5,5'-Tetramethylbenzidine) substrate was added. The enzyme activity was stopped using 100 µL of H_2_SO_4_ (1 N), and the optical density was read at 450 nm using an ELISA reader. GraphPad Prism Software v. 8.0.2 was used to perform the statistical analysis.

Immunophenotyping

CHO-K1 and CHO-K1-CD20 cells served as negative control and test groups, respectively. All the cells were synchronously cultured and treated with 1% Trypsin-EDTA (Bio-IDEA, Iran). The cells were then washed twice with PBS, and allophycocyanin-conjugated mouse antihuman CD20 antibody (IQ products, Netherlands) was utilized to stain cells, which were incubated at 4 °C for 1 h. After fixing the cells, they were analyzed using a flow cytometer (BD Biosciences, USA). Data analysis was performed using FlowJo analysis software version 7, 6, 1 (SABA Company, Tehran, Iran).

Single-cell cloning

Stable CHO-K1-CD20 pool was cultured in a puromycin-free culture medium for 30 passages. Single-cell cloning was performed by limiting dilution in 96-well plates. First, cells of the stable pool were cultured and counted after reaching 80% confluency. Then cells were seeded at a concentration of 0.5 cell/well in a total volume of 100 µL/well in antibiotic-free DMEM-F12 culture medium containing 20% FBS. After two weeks, single-cell clones were identified through microscopic observation and transferred to 24-well plates. Finally, six surviving clones were expanded and analyzed for CD20 expression.

## RESULTS

**Construction of the expression vector**

**Fig. 1:**
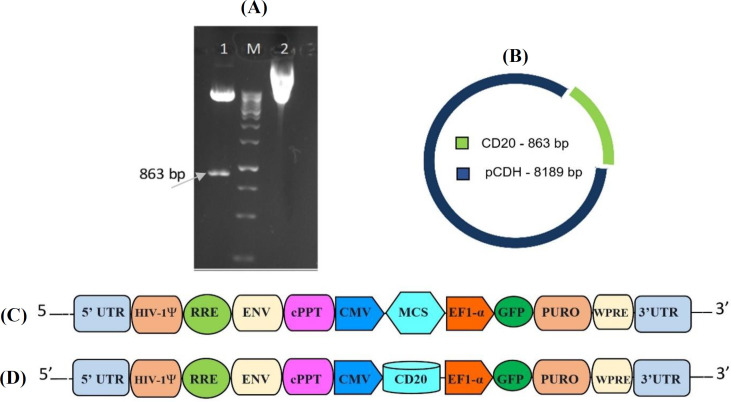
**(A)** Restriction pattern analysis of recombinant pCDH-CD20 vector. Lane 1, digested plasmid (using *Xba*I and *bam*HI restriction enzymes); lane 2, undigested plasmid; M, DNA size marker. **(**B**)** Schematic illustration of the transfer vector map indicating enzymes restriction sites. **(**C**)** Transfer vector pCDH-CMV-MCS-EF1-GFP-T2A-Puro. **(**D**)** Recombinant transfer vector pCDH containing *CD20* gene. Ψ, packaging signal; RRE, rev response element; ENV, envelope; cPPT, central polypurine tract; CMV, human cytomegalovirus major immediate early promoter/enhancer; EF1-α, human elongation factor-1a promoter; GFP, green fluorescent protein; PURO, puromycin; WPRE: woodchuck hepatitis virus post-transcriptional regulatory element; UTR, untranslated region

The recombinant vector constructed in this study, pCDH-CD20, is shown in Figure 1. Two specific primers (CD20 F and CD20 R) were designed based on the GenBank sequences (NM_021950.3) and used to amplify and clone CD20 fragment in the *Bam*HI and *Xba*I sites of the pCDH expression vector. Following PCR amplification, an 863-bp band corresponding to the full-length human CD20 antigen was observed. CD20 coding sequence was cloned into pCDH vector, downstream to the CMV promoter. A single band at 863 bp was identified in the restriction digestion analysis using *Bam*HI and *Xba*I. Also, 100% homology with NCBI databases was confirmed through the analysis of the cloned gene in pCDH vector, which indicated the successful cloning of the CD20 coding sequence.

**Construction of lentiviral particles**

**Fig. 2:**
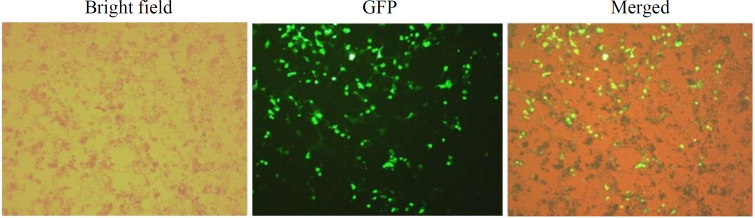
Pckaging HEK293T cells transduced with lentivirus-derived vectors. Analysis of fluorescent microscopy 24 hours post transfection affirmed the GFP expression in the packaging cells (scale bar =10 μm).

HEK293-T cells were used as the packaging cells. By co-transfection of these cells with recombinant LV transfer vector pCDH-CD20, together with psPAX2 and pMD2G plasmids, viral particles were produced. The integration of GFP gene in the genome of HEK293T transduced cells was evaluated 24 hours after transfection. Expression of GFP protein was visualized and corroborated after exposure to UV light by fluorescent microscopy (Fig. 2).

**Lentiviral transduction and development of the stable cell pool**

**Fig. 3:**
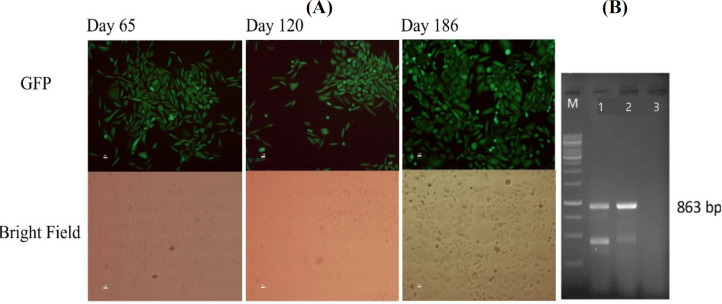
(A) Fluorescent images of GFP-positive stable polyclonal CHO-K1-CD20 cell. GFP expression evaluated up to three months using fluorescent microscopy (scale bar =10 μm). (B) Agarose gel electrophoresis of RT-PCR product. PCR product from stable pool showed a 863-bp band, confirming CD20 fragment. RT-PCR product: lane 1, stable cells; lane 2, positive control (pCDH-CD20 plasmid); lane 3, negative control; M, DNA ladder

Lentiviral particles were used for stable transduction of CHO-K1 cells. Ultracentrifugation has been utilized to concentrate viral particles^[^^35^^]^. In another study, a massive cell death was reported after using concentrated lentiviral particles^[^^37^^]^. Herein, we performed an optimized method for lentivirus preparation without using ultracentrifugation^[^^33^^]^. The lentivirus titer estimated at 1.2 × 10^7^ TU/mL following transduction of CHO-K1 cells at the multiplicity of infection of 1. The minimum killing concentration by puromycin selection was determined at 6 µgmL^-1^. GFP fluorescence was visualized in the transduced CHO-K1-CD20 cells. The expression of reporter GFP protein in the cell pool was analyzed continuously for a period of three months by fluorescent microscopy (Fig. 3A).

**Expression analysis in the stable cell pool**

***CD20 gene expression***

After antibiotic selection, the expression of CD20 mRNA was evaluated using RT-PCR analysis. The focus was on total RNA of the stable cell pool, as well as the untransfected CHO-K1 cells. Observation of the 863-bp band corresponding to the CD20 fragment confirmed the expression of CD20 gene in the stable cell pool (Fig. 3B).

***CD20 antigen expression***

**Fig. 4:**
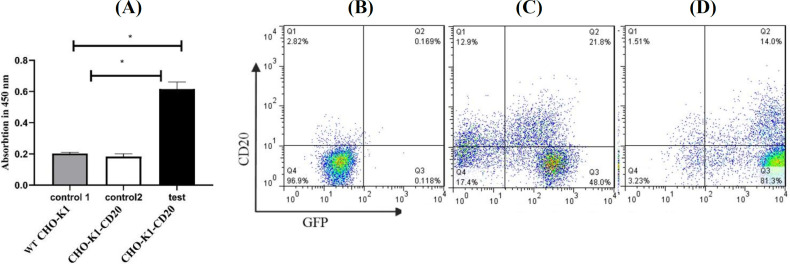
CD20 overexpression analysis in the stable polyclonal CHO-K1-CD20 pool transduced with lentivirus-derived vectors containing the sequence of copGFP and CD20. (A) Immunocytochemistry results. In control 1, wild-type (WT) CHO-K1 cells were treated with ofatumumab antibody, which has previously been expressed and purified in CHO cells, control 2 indicates CHO-K1-CD20 cells (stable polyclonal cell pool), which were treated with PBS, and test group shows CHO-K1-CD20 cells, which were treated with ofatumumab antibody. The indicated values are the mean of triplicate immunocytochemistry results for each experiment, and error bars represent standard deviation. Asterisks show a significant difference at p < 0.05. (B) CD20 expression analysis by flow cytometry in wild-type CHO-K1. (C) CD20 expression analysis by flow cytometry in the stable cell pool. (D) CD20 expression analysis by flow cytometry in stable cell pool after 30 passages. Q1, CD20^+^; Q2, CD20^+^ GFP^+^; Q3, GFP^+^; Q4, CD20^-^ GFP^-^

CHO-K1-CD20 cell pool was cultured in the puromycin-free medium for approximately five passages before the evaluation of CD20 expression. The results obtained from immunocytochemistry (Fig. 4A) confirmed that CD20 protein was expressed in the test group. Besides, according to immunophenotyping analysis (Fig. 4), nearly 35% of cell pool expressed CD20. Neither GFP nor CD20 expression was observed in the wild-type (un-transfected) CHO-K1 cells. We also repeated immunophenotyping examination for the stable cell pool after culturing in the antibiotic-free culture medium for 30 passages to evaluate the effects of multiple passages on the expression of the target proteins. The decrease in the percentage of the CD20-expressing cells (Fig. 4) indicated that 16% of the cells in the pool expressed CD20.

**Analysis of CD20 expression in single-cell clones**

**Fig. 5:**
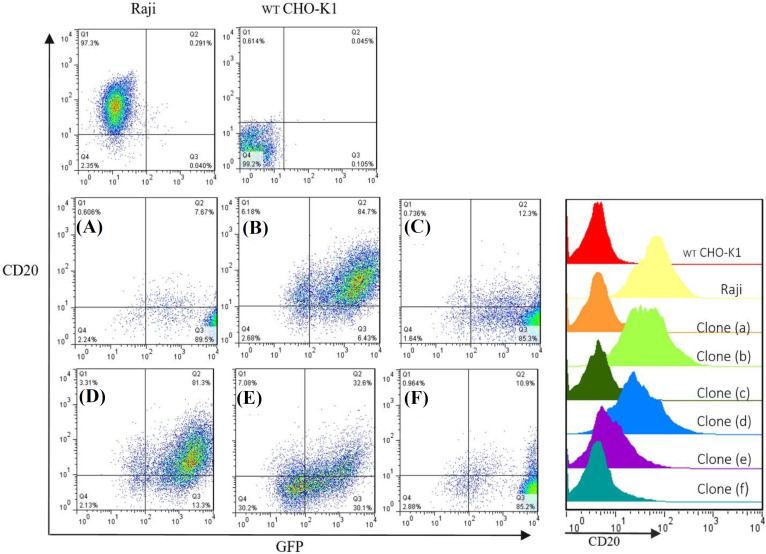
**CD20 expression analysis in stable monoclonal CHO-K1-CD20 clones transduced with lentivirus-derived vectors containing the sequence of copGFP and CD20 following 30 passages. Quadrant statistics are provided in each dot plot. Q1, CD20** ^+^ **; Q2, CD20** ^+^ **GFP** ^+^ **; Q3, GFP+; Q4, CD20- GFP-. (A) CD20 expression was evaluated in Raji and WT CHO-K1 cells as positive and negative controls, respectively. (A-F) Immunophenotyping analysis in expanded monoclonal CHO-K1-CD20 cell clones** **showing** **different levels of CD20 antigen. Clones (b) and (d) with the highest percentage of CD20-expressing cells and mean fluorescence intensity considered as high CD20-expressing clones. Forty percentage of cells in clone (e) show CD20 antigen expression and is considered as a medium CD20-expressing clone. In nearly 10% of cells in clones a, c, and f, CD20 antigen was detected, and these populations ** **considered as low expressing clones. Histogram overlay indicates CD20-allophycocyanin mean fluorescence intensity of eight independent experiments performed for control cell populations and single-cell clones. Histograms were presented using FlowJo software**

Stable single-cell clones were constructed out of the stable cell pool by serial dilution cloning in order to reach clones with different levels of CD20 expression. Six GFP-positive monoclonal clones were selected, cultured and expanded. Immunophenotyping was employed for the analysis of CD20 expression in the six clones. We conducted immunophenotyping for the clonal populations after their culture in the antibiotic-free culture medium for three months (at least 30 passages), to evaluate the stability of the protein expression following multiple passages. The Raji cells (malignant B lymphocytes), which expose the CD20 antigen, were used as positive control. Figure 5 illustrates CD20 expression analysis in the stable CHO-K1-CD20 clones. Clones with different CD20 antigen expression levels considered as high, medium and low CD20-expressing clones and stored for further investigations.

## DISCUSSION

The second-generation lentiviral vector was used in this study for the generation of CD20-expressing stable non-hematopoietic cell lines. The established system, however, is not limited to the CD20 expression and is applicable for the generation of various in vitro models based on CHO cells. The vector harbors the transgene with an upstream CMV promoter, as well as GFP and puromycin genes, of which both were under the control of the EF-1α promoter. Simultaneous expression of CD20 with GFP protein facilitates the detection of CD20-expressing cells and clones. We successfully stabilized the expression of CD20 for more than three months, and during this period, the cells were regularly observed with fluorescent microscopy for the detection of GFP protein. After 30 passages of the polyclonal stable pool, expression of CD20 antigen decreased while mean GFP-specific fluorescence intensity increased. Single-cell cloning was performed to achieve clones with different CD20-expression levels, followed by the analysis of protein expression in the expanded clones for three months. We observed various expression levels in monoclonal populations. Immunophenotyping results demonstrated that two pools had the highest level of expression for both GFP and CD20, while other pools had approximately 10% CD20-expressing cells and relatively more than 95% GFP-positive cells. These results can be attributed to the difference between the promoters controlling GFP and CD20. Other studies reported higher percentages of GFP-positive cells and improved mean GFP fluorescence intensity when EF-1α promoter compared to the CMV promoter^[^^38^^]^. In fact, low level transgene expression could be attributed to multi-site methylation of CMV promoter^[^^39^^]^. The purpose of this study was to establish the stable non-hematopoietic CD20-expressing cell lines. 

While the establishment of stable cell lines overcomes some drawbacks related to transient transfection, it is still a tedious procedure. Lentiviral-based gene delivery system, however, offers several advantageous including the speed and convenience^[^^40^^]^. High transduction efficiency and long-lasting expression of the transgene are hallmarks of this system^[^^13^^,^^24^^]^. Recently, lentiviruses have been exploited for large-scale protein expression with various purposes. For instance, Elegheert and co-workers^[^^40^^]^ introduced a specifically designed transfer plasmid (pHR-CMV-TetO_2_) for rapid and scalable protein expression in HEK293 cell lines, resulting in the fast (seven days) generation of polyclonal cell lines, with the lead-time for protein production of nearly 3-4 weeks. Compared to transient transfection, these researchers recorded a 3-10-fold increase in protein production yield per cell. Besides, scalable production of different antigens for the diagnostic purposes or vaccine development is of high importance. More recently, Zhang et al.^[^^41^^]^ have developed stable HEK293 cell pools transduced with lentiviruses for the large-scale production of classical swine fever virus E2 protein. They reached 2 g/L titer of E2 protein in semi-perfusion culture. Endowing with a desirable integration competency, lentiviral vectors facilitate transgene cassette delivery without affecting endogenous gene expression of the host cells^[^^28^^]^. Nowadays, lentivirus vectors are considered as safe and efficient gene transfer vehicles for cell and gene therapy applications. In this regard, the experimental model of ex vivo gene modification for hematopoietic gene therapy revealed that the population of human CD34^+^ cells transduced with lentiviral vectors remained extremely similar at the phenotypic level^[^^42^^]^. Although lentiviruses have a potential risk for insertional mutagenesis and oncogenicity, there is no reported evidence regarding these concerns using wild-type HIV and lentiviral vectors based on this virus^[^^43^^]^. They also harbor long terminal repeats with low basal and inducible promoter activity^[^^44^^,^^45^^]^. The engineering and preparation process of lentiviral vectors are commonly more sophisticated than non-viral methods; however, virus-based transduction is highly efficient, especially in case of hard-to-transfect and primary cells^[^^46^^]^. Recombinant complex proteins such as antibodies and membrane protein markers are mostly produced in mammalian host systems such as CHO cells, revealing much stability and efficiency in transfection and post-translational processing in the secretory pathway, which is pivotal for the correct folding of intricate proteins^[^^47^^,^^48^^]^. In line with our study, Tati et al.^[^^49^^]^ cloned and delivered the human CD52 gene into the CHO-K1 cells using the electroporation method. Hedayatizadeh-Omran and colleagues^[^^50^^]^ have also established a recombinant HER2-expressing CHO cell line as an in vitro model for breast cancer and HER-related studies.

We confirmed stable expression of CD20 in the CHO-K1 cell line using lentiviral-based gene delivery method. To our knowledge, this is the first report on using second-generation lentiviral vectors for the establishment of a non-hematopoietic cell-based system, which permanently expresses full-length human CD20 antigen. The introduced in vitro model can be used in experimental settings involving CD20 antigen and/or anti-CD therapeutics. Moreover, it can be applicable for mAb-CD20 internalization assays, as well as CD20 ligand screening and CD20 binding assays. Clones expanded in this study represented different levels of CD20 expression, including 82%, 50%, and 10%, which were classified as high, medium and low expressing clones, respectively. The resulting clones can be utilized in the assays to mimic various disease stages with different levels of cell surface CD20 antigen.

## DECLARATIONS

### Acknowledgments

The authors would like to thank Prof. Eberhard Korsching for his substantial contribution toward the revision and editing the manuscript. This article has been extracted from the thesis written by Ms. Niloufar Mohammadkhani in School of Medicine Shahid Beheshti University of Medical Sciences, Tehran, Iran (registration no. 317 M). NM created graphical abstract with *BioRender.com.*

### Ethical statement

This article does not contain any studies involving animals performed by any of the authors. The CLL patient agreed to participate in this study and his blood sample was obtained with informed consent.

### Data availability

The analyzed data sets generated during the study are available from the corresponding author on reasonable request. 

### Author contributions

NM: performed laboratory assessments, wrote the manuscript and contributed to conceptualization; MR: contributed in conceptualization and supervised the project. AR and RH: contributed in study design, data analysis. All authors cooperated in scientific discussion and data interpretation, and contributed to revising and editing the original draft and approved the final manuscript.

### Conflict of interest

None declared.

### Funding/support

The authors received no financial support for the research and authorship of this article.

## Supplementary materials

**Supplementary Figure 1:**
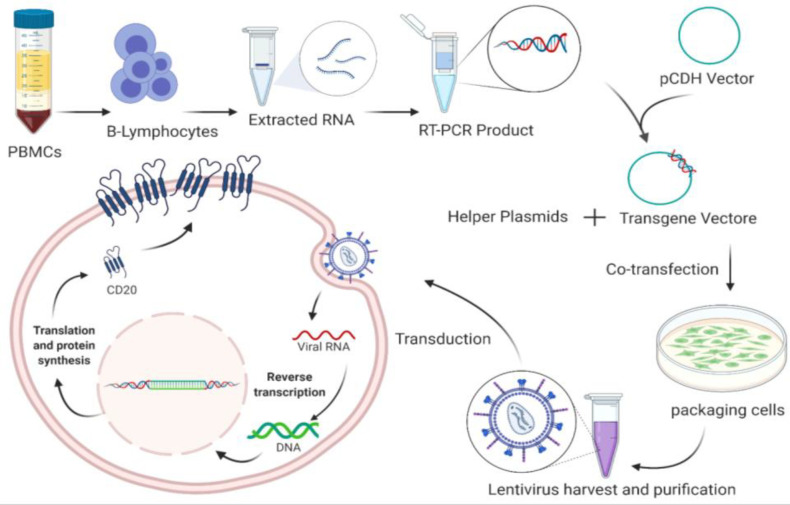
Schematic representation of the study.
